# Necrotizing Scleritis Post Cataract Surgery: A Diagnostic Dilemma

**DOI:** 10.7759/cureus.70861

**Published:** 2024-10-04

**Authors:** Vishakha Vatkar, Deepaswi Bhavsar, Tushar Agrawal, Pradipta P Potdar, Kalibo Jakhalu

**Affiliations:** 1 Ophthalmology, Dr. D.Y. Patil Medical College, Hospital and Research Centre, Dr. D.Y. Patil Vidyapeeth (Deemed to be University), Pune, IND

**Keywords:** delayed hypersensitivity reaction, necrotising scleritis, ophthalmology, small-incision cataract surgery, surgically-induced necrotizing scleritis

## Abstract

Scleral necrosis following ocular surgery can lead to significant ocular and systemic complications. The two most prevalent procedures associated with surgically-induced scleral necrosis are pterygium excision and cataract surgery. This condition represents a rare delayed hypersensitivity reaction. We present a case of a male patient in his late 60s from India who experienced progressive scleral necrosis in his right eye six months after undergoing small-incision cataract surgery (SICS). The patient exhibited pain and redness without any decline in vision, and all systemic evaluations returned normal results. Ultimately, he was scheduled for a scleral patch graft.

This case emphasizes the necessity for rapid diagnosis of surgically-induced necrotizing scleritis (SINS) and timely intervention.

## Introduction

Surgically-induced necrotizing scleritis (SINS) is an uncommon autoimmune response affecting the sclera at the site of previous surgical incisions [1]. Its incidence is reported to be extremely low, often cited as less than one in 10,000 cases, with some estimates suggesting it may be even rarer, at fewer than one in 100,000 [2]. This condition is more frequently observed in women, typically occurring in individuals during their fifth decade of life [3]. Surgically-induced necrotizing scleritis has been documented following various procedures, including cataract and pterygium surgeries, strabismus surgery, and trabeculectomy. The pathogenesis generally involves ocular trauma as a triggering factor that leads to scleral necrosis, influenced by unusual local and systemic factors [4]. In this case report, we present a patient who developed SINS six months after cataract surgery, resulting in a diagnostic challenge.

## Case presentation

A male in his late 60s came to the ophthalmology outpatient department with complaints of sudden pain and redness in his right eye six months after small-incision cataract surgery (SICS). The surgery included the placement of a 6 mm rigid polymethyl methacrylate (PMMA) intra-ocular lens. His ocular history included left eye cataract surgery done one year back by the same surgeon in the same hospital, while the right eye was operated on for mature cataract through a 6.5 mm scleral incision. Intra-operatively, three interrupted sutures were taken at the incision site. He was asymptomatic for five months after surgery. His medical history included uncontrolled diabetes with neuropathic and nephropathic complications. He also had a history of cardiac surgery.

**Figure 1:**
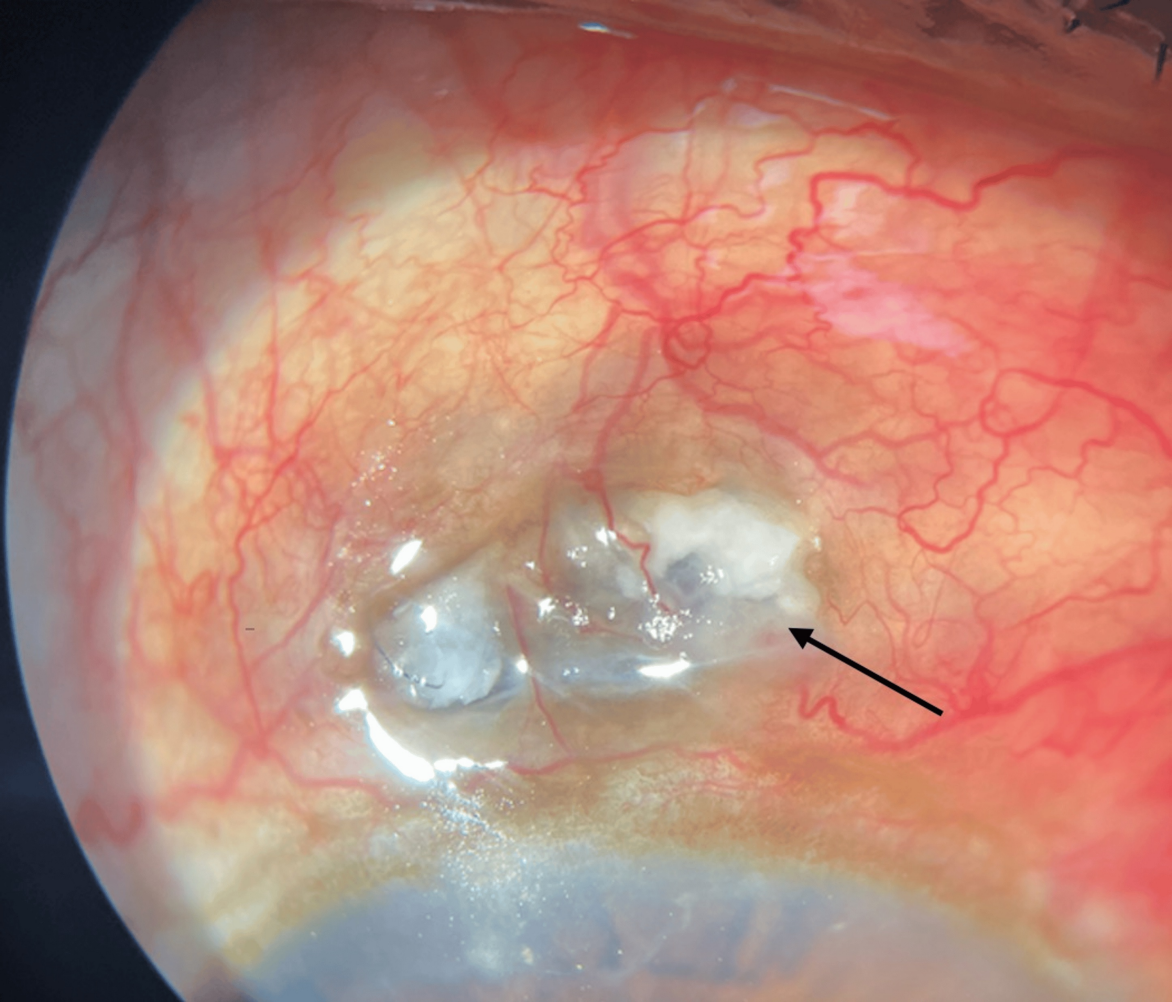
Anterior segment photograph of the right eye showing circumcorneal congestion with scleral thinning and tortuous vessels

On examination, the best corrected visual acuity (BCVA) was 20/32 in the right eye and 20/20 in the left eye, with near vision N6 in both. Intraocular pressures were recorded at 16 mm Hg in the right eye and 14 mm Hg in the left. Slit lamp examination revealed generalized conjunctival hyperemia, circumcorneal congestion, and episcleral and scleral congestion with tortuous vessels at the incision site, measuring 4x6 mm in the right eye (Figure 1).

**Figure 2:**
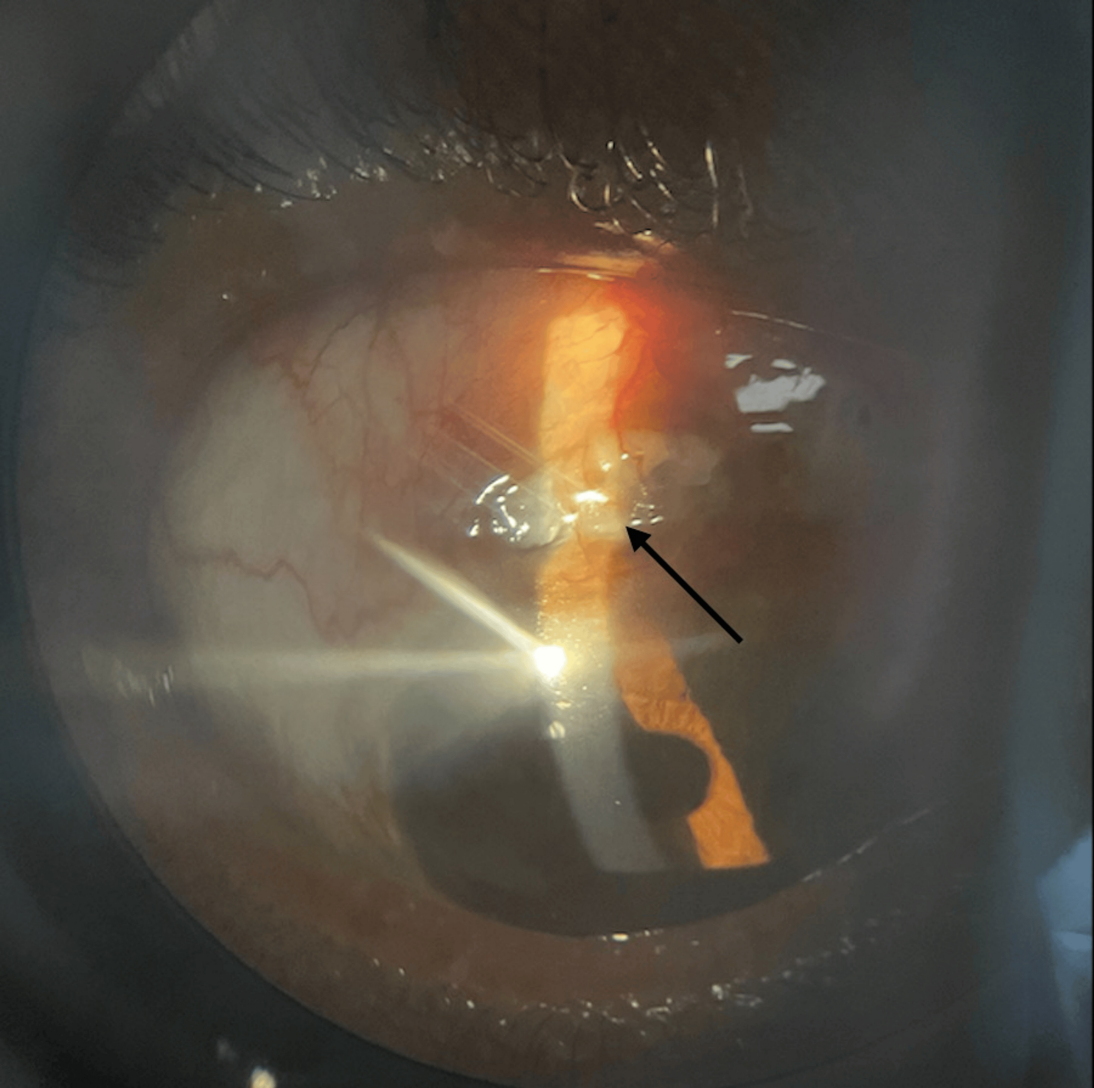
Right eye showing diffuse congestion with scleral necrosis and thinning through slit view

Scleral thinning was also noticed in the right eye (Figure 2).

**Figure 3:**
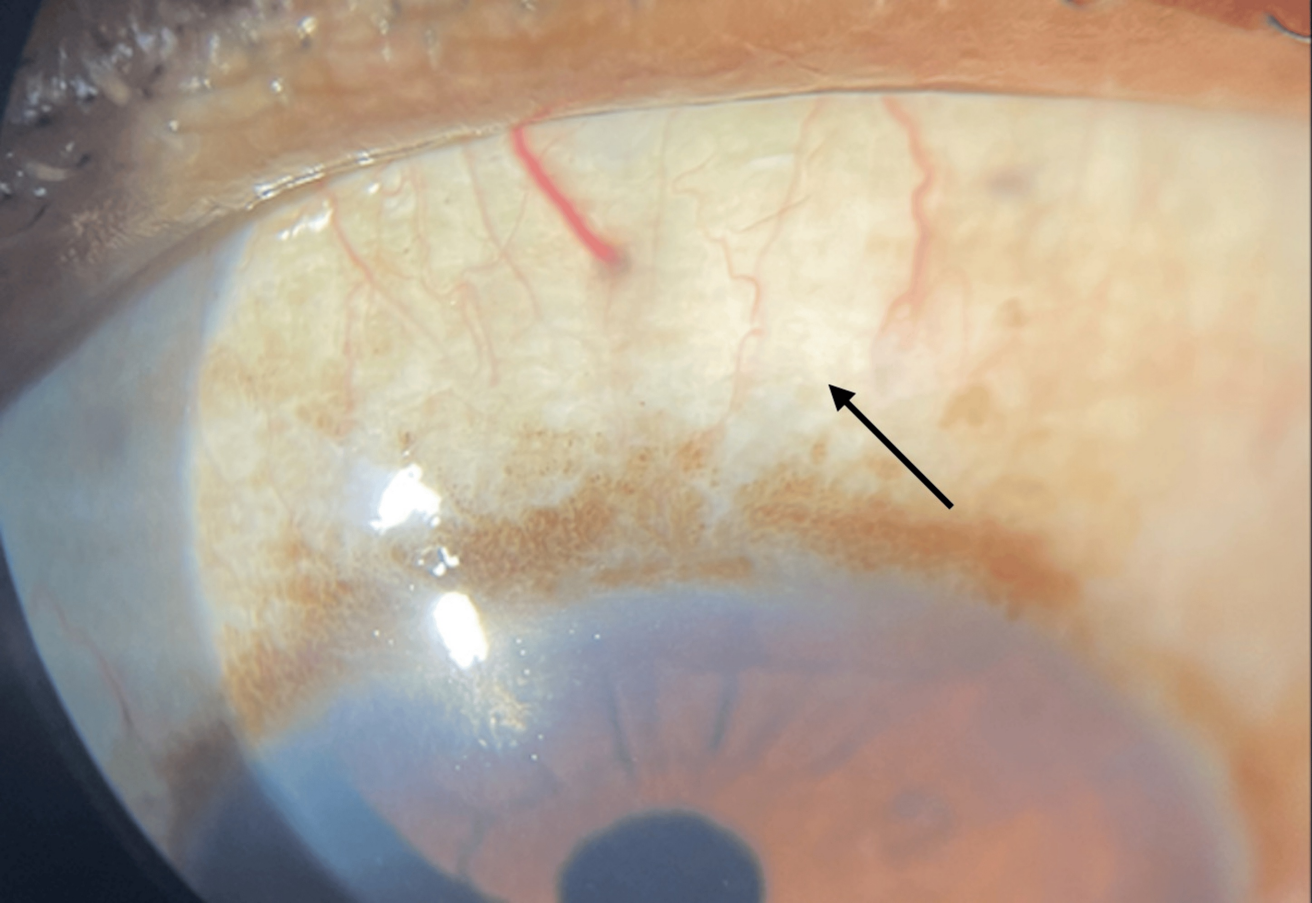
Left eye showing normal appearing sclera post small-incision cataract surgery

The anterior segment of the left eye was within normal limits (Figure 3).

Fundus examination of both eyes revealed mild non-proliferative diabetic retinopathy, with no signs of macular edema. Based on the clinical findings, anterior necrotizing scleritis was diagnosed, though it remained uncertain whether the condition was due to an infection or related to previous surgery. The initial treatment regimen included prednisolone eye drops every six hours and moxifloxacin 0.5% eye drops administered four times daily. A thorough systemic evaluation was performed, which encompassed a complete blood count, biochemical analysis, and serological tests for HLA-B27, HLA-B51, and HLA-A29, along with autoantibody testing, rheumatoid factor, and C-reactive protein. Additional assessments included a tuberculin skin test and a chest X-ray. Both polymerase chain reaction (PCR) analysis of conjunctival scraping and an aqueous humor sample for microbiological culture returned negative results. Despite antibiotic treatment, the patient's condition worsened, necessitating scleral patch graft surgery. Unfortunately, the patient was lost to follow-up due to financial constraints.

## Discussion

Surgically-induced necrotizing scleritis is a rare yet serious complication following ocular surgery, which can occur anywhere from the first postoperative day to several years later. This condition presents diagnostic challenges, as scleral thinning may be overlooked. Patients may initially report mild discomfort and conjunctival congestion, which can be mistaken for dry eye or conjunctivitis, leading to delays in treatment. In advanced cases, significant visual impairment, scleral necrosis, anterior staphylomas, and peripheral corneal ulcers may develop. To rule out infectious causes, it is crucial to perform a scleral biopsy and intraocular samples for PCR analysis and wound scraping for microbiological culture. Given the potential impact on vision, early intervention is vital. Diabetes mellitus may exacerbate this condition by creating a pro-ischemic inflammatory environment [5].

Oral prednisolone is often considered the first-line treatment for non-infectious necrotizing scleritis, while intravenous methylprednisolone may be used in severe cases. Additional therapies may include non-steroidal immunotherapy, monoclonal antibodies against tumor necrosis factor-alpha, interleukin-6 inhibitors, and anti-CD20 targeted drugs. For cases with progressive scleral necrosis, surgical options such as conjunctival debridement, conjunctival and Tenon's flap grafts, amniotic membrane transplantation, and scleral patch grafts may be necessary [6].

Mártires et al. documented a rare instance of SINS following intraocular lens replacement, emphasizing the importance of early recognition and treatment. The combination of systemic corticosteroids and scleral patch grafting effectively managed the condition, leading to symptom relief and partial visual recovery [3]. Das et al. provided insights into the presentation and management of postoperative necrotizing scleritis, demonstrating the necessity of prompt diagnosis and intervention. Their findings underscored the variability in patient outcomes, highlighting the need for tailored treatment plans and ongoing monitoring [7].

## Conclusions

Inadequate pre-operative assessment for any type of ocular surgery can lead to SINS, making it a challenging condition to diagnose, prevent, and manage. Factors such as previous ocular surgery, excessive cautery use, extensive surgical manipulation, associated systemic autoimmune disorders, and toxic adjunctive therapies require careful consideration. Scleral inflammation can be both self-limiting and vision-threatening, complicating the diagnosis of SINS. Further research and large randomized controlled trials on postoperative scleral necrosis are necessary to identify contributing factors, at-risk patients, and the safety of various medical and surgical interventions for this condition.
